# High dietary zinc supplementation increases the occurrence of tetracycline and sulfonamide resistance genes in the intestine of weaned pigs

**DOI:** 10.1186/s13099-015-0071-3

**Published:** 2015-08-26

**Authors:** Wilfried Vahjen, Dominika Pietruszyńska, Ingo C. Starke, Jürgen Zentek

**Affiliations:** Institute of Animal Nutrition, Freie Universität Berlin, Berlin, Germany

**Keywords:** Antibiotic resistance, Pig, Sulfonamide, Tetracycline, Zinc oxide

## Abstract

**Background:**

Dietary zinc oxide is used in pig nutrition to combat post weaning diarrhoea. Recent data suggests that high doses (2.5 g/kg feed) increase the bacterial antibiotic resistance development in weaned pigs. Therefore, the aim of this study was to investigate the development of enterobacterial antibiotic resistance genes in the intestinal tract of weaned pigs.

**Findings:**

Weaned pigs were fed diets for 4 weeks containing 57 (low), 164 (intermediate) or 2425 (high) mg kg^−1^ analytical grade ZnO. DNA extracts from stomach, mid-jejunum, terminal ileum and colon ascendens were amplified by qPCR assays to quantify copy numbers for the tetracycline (*tet*A) and sulfonamide (*sul*1) resistance genes in Gram-negative bacteria. Overall, the combined data (n = 336) showed that copy numbers for tetracycline and sulfonamide resistance genes were significantly increased in the high zinc treatment compared to the low (*tet*A: *p* value < 10^−6^; *sul*1: p value = 1 × 10^−5^) or intermediate (*tet*A: P < 1.6 × 10^−4^; *sul*1: P = 3.2 × 10^−4^) zinc treatment. Regarding the time dependent development, no treatment effects were seen 1 week after weaning, but significant differences between high and low/intermediate zinc treatments evolved 2 weeks after weaning. The increased number of *tet*A and *sul*1 copies was not confined to the hind gut, but was already present in stomach contents.

**Conclusions:**

The results of this study suggest that the use of high doses of dietary zinc beyond 2 weeks after weaning should be avoided in pigs due to the possible increase of antibiotic resistance in Gram-negative bacteria.

**Electronic supplementary material:**

The online version of this article (doi:10.1186/s13099-015-0071-3) contains supplementary material, which is available to authorized users.

## Background

In many countries high doses (up to 3000 mg/kg feed) of dietary zinc oxide are used in pig nutrition to successfully combat post weaning diarrhea in pigs, which is often induced by pathogenic *Escherichia coli* strains [1]. It has been shown that the intestinal microbiota of freshly weaned pigs is strongly perturbed by such high dietary zinc doses [2]. However, a direct inhibition of total enterobacteria or coliforms was not observed in several studies on this topic [3–5] and there are even indications that high dietary zinc may increase the diversity of *Enterobacteriaceae* [6]. Possibly connected to the hypothesis that dietary zinc enhances enterobacterial diversity is a recent report on the increased occurrence of multi-resistant *E. coli* strains in pigs fed high zinc doses [7]. Although intrinsic resistances against certain antibiotics are known for Gram-negative bacteria, the lack of antibiotic use in the mentioned studies makes it much more likely that indeed zinc oxide is the determining factor for the observed increase in antibiotic resistances. These results are disconcerting, as enterobacteria are known to exchange genetic information frequently [8, 9]. An increased spread of antibiotic resistance genes via horizontal gene transfer within the *Enterobacteriaceae* could lead to the emergence of strains with high virulence.

The work by Bednorz et al. [7] showed a drastic zinc related increase (18.6 %) especially for tetracycline and sulfonamide resistant *E. coli* strains. The present study expands our knowledge on the relation between dietary zinc and antibiotic resistance by quantifying the occurrence of the tetracycline (*tet*A) and sulfonamide (*sul*1) genes of Gram-negative bacteria regarding development and distribution of these genes in the intestinal tract of weaned pigs fed different amounts of dietary zinc during 6, 13, 20 or 27 days post-weaning.

## Methods

The study was conducted according to the German Animal Welfare Act (TierSchG) and approved by the local state office of occupational health and technical safety ‘Landesamt für Gesundheit und Soziales, Berlin’ (LaGeSo Reg. Nr. 0347/09). A total of 84 purebred German Landrace piglets (7.2 ± 1.2 kg) from 10 sows of our Institute animal husbandry were weaned at 26 ± 1 days of age. Neither sows nor piglets received any antibiotic treatment. Sows and their offspring were kept in flatdeck pens in the same stable, the stable and pens were cleaned daily. No animal showed signs of disease during the trial period. From 12 days of age, piglets had access to a commercial non-medicated prestarter diet which was formulated to meet requirements of piglets with average body weight of 5–10 kg. After weaning, animals were randomly assigned to three treatment groups, kept in flatdeck pens with two animals per pen Weaned pigs were fed an antibiotic free commercial starter diet containing low (57 mg/kg), intermediate (164 mg/kg) or high (2,425 mg/kg) zinc as analytical grade zinc oxide (Sigma Aldrich, Taufkirchen, Germany) to cover minimal to excessive dietary zinc supplementation.

On days 32, 39, 46 and 53 of life, seven pigs per group were euthanized 4 h after their last meal. The piglets were sedated with 20 mg/kg BW of ketamine hydrochloride (Ursotamin^®^, Serumwerk Bernburg AG, Germany) and 2 mg/kg BW of azaperone (Stresnil^®^, Jansen-Cilag, Neuss, Germany) prior to euthanasia with intracardial injection of 10 mg/kg BW of tetracaine hydrochloride, mebezonium iodide and embutramide (T61^®^, Intervet, Unterschleißheim, Germany). Digesta contents were immediately shock-frozen in liquid nitrogen and stored at −80 °C until further processing.

DNA extraction was performed with a commercial kit (Qiagen Stool kit, Qiagen, Hilden, Germany) with 200 mg digesta according to the instructions of the manufacture except for an increase in temperature to 90 °C during the lysis step. DNA content in the extracts was quantified by fluorometric determination using a fluorospectrometer (ND3300, Thermo Scientific, Henningsdorf, Germany). DNA concentration was adjusted to 50 ng/µL before PCR amplification.

Primers against *tet*A (tetA-1f: 5′-ATCATGCAACTCGTCGGACA-3′; tetA1r: 5′-TCGTGTCCCAATGAAAGCGA-3′) and *sul*1 (sul1-3f: 5′-CGATCCGGGGATGGGATTTT-3′; sul1-3r: 5′-CACCGAGACCAATAGCGGAA-3′) for quantitative realtime PCR detection were generated from published sequences and validated against DNA from a range of positive and negative reference strains (see Additional file 1: Table S1). All primers were purchased from MWG Biotech (Straubing, Germany). A Stratagene MX3000p (Stratagene, Amsterdam, The Netherlands) was used for PCR amplification and fluorescent data collection. The master mix consisted of 12.5 µL Brilliant II SYBR^®^ Green QPCR Master Mix with Low ROX (Stratagene, Amsterdam, Netherlands), 0.5 µL of each primer (10 µM) and 10.5 µL water. One microliter sample extract was added before PCR amplification (1 × 10 min at 95 °C followed by 40× 30 s at 95 °C, 60 s at 60 °C and 60 s at 72 °C). A melting curve was generated to confirm correct PCR amplification. Dilution factors for each individual DNA extract were used to calculate copy number/g based on total DNA amount in the 200 mg sample.

Statistical analysis was carried out with SPSS 19.0 (SPSS Inc., IL, USA). The data was structured to address questions on overall effect, time effects as well as intestinal site. Thus, the data was grouped for overall effect (combining all days and all intestinal site data), grouped for time effects (data split into sampling day combining all intestinal sites) as well as for effects of intestinal site (data split into intestinal site combining all sampling days). Significant differences were determined by the Kruskal–Wallis test followed by the Mann–Whitney U test, where appropriate. Differences at *P* < 0.05 were considered significant.

## Results and discussion

This study was part of a larger feeding trial in which the impact of zinc oxide on microbiological [2, 3, 6, 7], morphological and immunological [10] as well as physiological [11] parameters were investigated. The interested reader is referred to these publications for a deeper analysis on the subject.

Overall, the combined data (n = 336) showed a significant increase of copy numbers for *tet*A and *sul*1 in the high zinc treatment compared to the low or intermediate zinc treatment (Table 1). This result is in line with data for tetracycline and sulfonamide resistant *E. coli* strains by Bednorz et al. [7]. Thus, this overall analysis shows that high doses of dietary zinc oxide increase tetracycline and sulfonamide resistance genes of Gram-negative bacteria in weaned pigs. The possible connection between zinc- and antibiotic resistance has already been noted for *E. coli* isolates from swine manure [12] and also for multi resistant *Staphylococcus aureus* isolates of porcine origin [13].

**Table 1:** Overall effect of dietary zinc on the occurrence of *tet*A and *sul*1 genes in pigs (n = 336)

Dietary Zn	Antibiotic resistance genes [log copy number/g sample]	
	tetA	sul1
Low Zn	4.78 (±0.63)^a^	5.43 (±0.63)^a^
Intermediate Zn	4.94 (±0.59)^a^	5.50 (±0.69)^a^
High Zn	5.29 (±0.60)^b^	5.82 (±0.52)^b^

Sampling days: 6, 13, 20 or 27 days post-weaning (26 days); intestinal sites: stomach, mid-jejunum, terminal ileum, colon ascendens

^a,b^Significantly different within a column (p ≤ 0.05; Mann–Whitney U test)

**Fig. 1:**
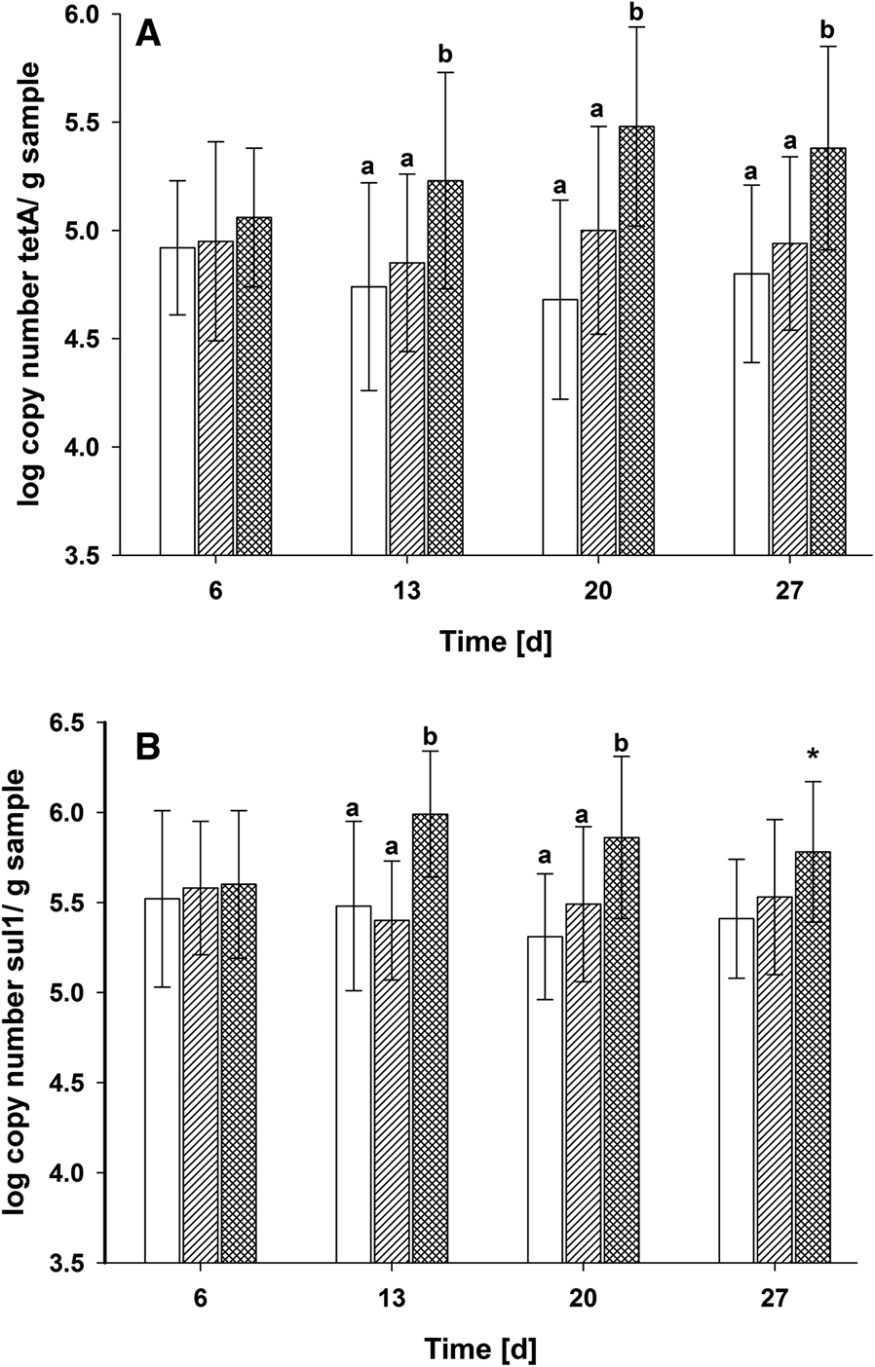
Time dependent effect of antibiotic resistance development. Combined data from all intestinal sites; *open bar* low dietary Zn; *bar* *with diagonal lines* intermediate dietary Zn; *hatched bar* high dietary Zn; ^a,b^Significantly different; ^*^ = trend (p < 0.1) for difference to low and intermediate dietary Zn groups. **a** *tet*A gene; **b** *sul*1 gene

The occurrence of *tet*A and *sul*1 was time dependent (Fig. 1). No treatment effects were observed 1 week after weaning, but significant differences between low/intermediate and high zinc treatment groups evolved 2 weeks (*sul*1) and 3 weeks (*tet*A) after weaning and remained on a higher level until the end of the trial (see also Additional file 1: Table S2). Considering the reduction of enterobacteria in the intestine during the post weaning phase in pigs [2], it can be expected that the ratio of antibiotic resistance carrying enterobacteria even increased. An ex vivo study with stomach and jejunum samples from the same animals has shown also that bacterial zinc resistance develops 2 week after weaning [14]. Furthermore, in that study enterobacteria were much less influenced by high zinc doses than other bacterial groups like clostridia and especially lactobacilli. Therefore, the lack of significant differences between diets 1 week after weaning may reflect a coupled development of zinc and antibiotic resistance over time. This has implications for the extent of dietary zinc supplementation. Although it is recognized that the main beneficial results occur up to 2 weeks after weaning [1], commercial use of high dietary zinc extends beyond this time frame, especially in Asia and the Americas [15–17].

The antibiotic resistance genes *tet*A and *sul*1 differ in their modes of action. Resistance against tetracycline in the form of the *tet*A gene is active, because it leads to the expression of a membrane bound efflux protein that recognizes tetracycline [18]. On the other hand, sulfonamide resistance (*sul*1 gene) is passive as resistant bacteria develop sulfonamide insensitive dihydropteroate synthases [19]. Thus, different modes of action may have occurred for the antibiotic resistance gene development in the intestine of pigs. An increase of antibiotic resistance due to the presence of heavy metals such as Hg, Cd, Cu and Zn can take place via co-resistance or cross-resistance [20]. Co-resistance is defined as the close proximity of two or more genetic elements encoding for resistances, while cross-resistance evolves when an antibacterial agent attacks the same target, for instance efflux systems that simultaneously transport two or more types of antibacterial agents [21].

In the case of tetracycline resistance, a cross-resistance mechanism may apply here, as zinc resistant strains would also expel tetracycline using the same efflux system. This is known for some bacteria regarding zinc and tetracycline resistance [22]. Sulfonamide resistance would rather follow the co-resistance path, as sulfonamides act as competitors of 4-aminobenzoate which is one of the substrates of dihydropteroate synthases. The enzyme is not known to be inhibited by zinc; on the contrary, it is an enzyme with zinc as cofactor [23]. Therefore, it is unlikely that zinc itself would have any influence on the development of sulfonamide resistance. Here, an increase via horizontal gene transfer is more likely the reason for an increased number of *sul*1 genes. This hypothesis was also put forward by Bednorz et al. [7]. Thus, multiple mechanisms may be involved in the development of antibiotic resistance in the porcine intestine due to the presence of zinc.

**Fig. 2:**
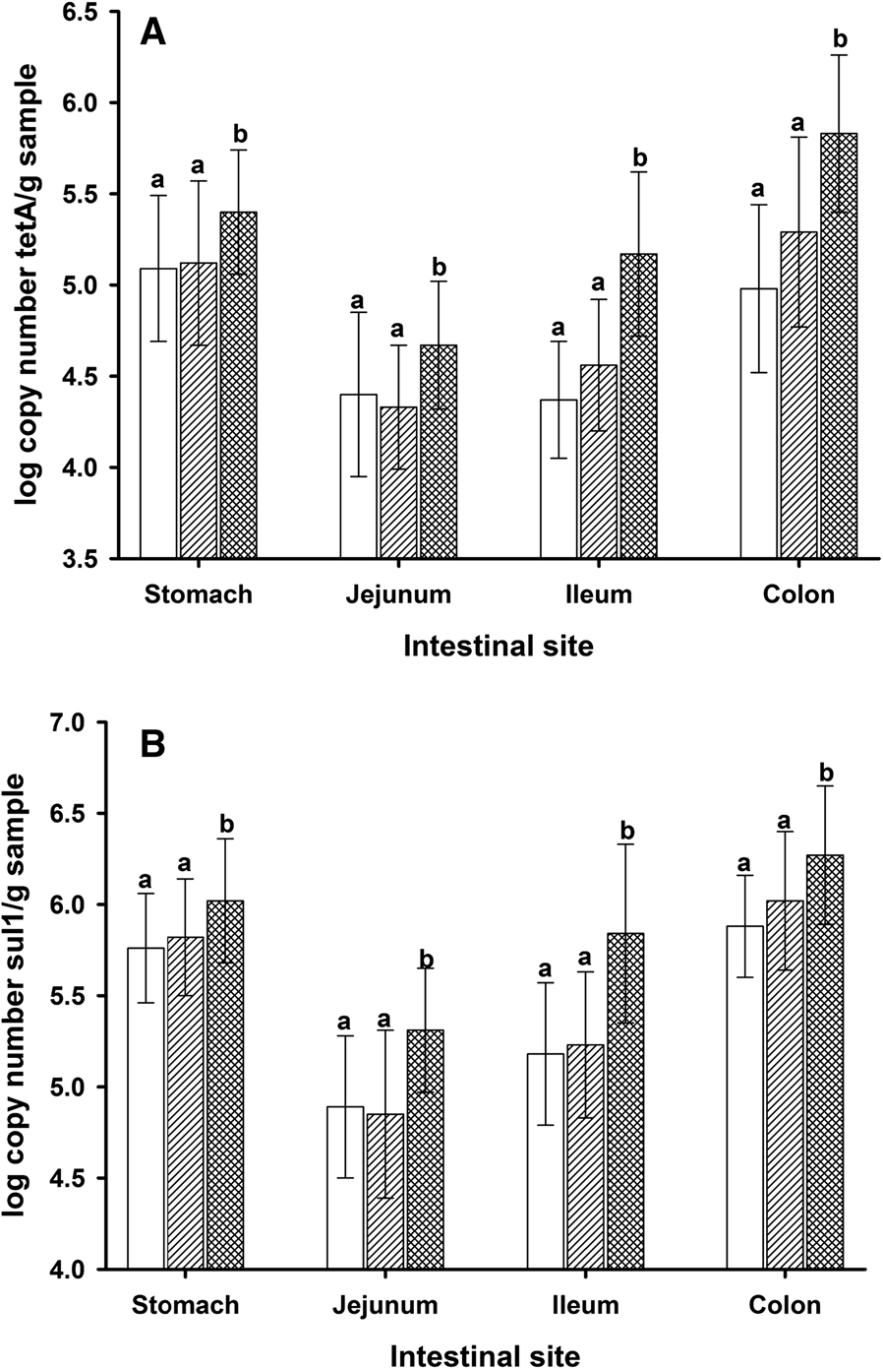
Distribution of antibiotic resistance in the intestine of pigs. Combined data from all sampling days; *open bar* low dietary Zn; *bar* *with diagonal lines* intermediate dietary Zn; *hatched* *bar* high dietary Zn; ^a,b^Significantly different; **a** = *tet*A gene; **b** *sul*1 gene

The presence of *tet*A and *sul*1 genes within the gastro-intestinal tract was also addressed in this study. An analysis for gastro-intestinal sites revealed that significant differences between low/intermediate and high treatment groups occurred already in the stomach and continued throughout the intestinal tract (Fig. 2). The lowest copy numbers for each group were found in the small intestine. Pigs harbor a very metabolically active gastric microbiota during the first weeks after weaning, due to a comparably high pH in the stomach. Enterobacteria and even strict anaerobic bacterial groups can be found via molecular methods in the stomach and small intestine [2, 24]. A hypothesis put forward by our research group [3, 14] states that zinc ions are released already in the stomach, thereby modifying the microbiota that reaches the small and large intestine, because zinc oxide displays increased solubility at acidic pH. Indeed, the ratio of free zinc ions to total zinc is highest in the stomach, followed by reduced ratios in the small intestine, while only very low concentrations of free zinc are observed in the large intestine [25]. Thus, high concentrations of dietary zinc may have the strongest impact in the stomach, generating zinc resistant enterobacteria which then “inoculate” the small and large intestine.

## Conclusions

This study has shown that high doses of dietary zinc increase the number of *tet*A and *sul*1 gene copies in the gastrointestinal tract of pigs. Further studies on this topic are necessary to elucidate the molecular mechanisms and time frame of antibiotic resistance development. However, due to the time dependent development a use of high doses of dietary zinc beyond 2 weeks after weaning should be avoided in pigs because of the possible increase of antibiotic resistance in Gram-negative bacteria.

## Additional file

**Additional file 1.** Strains used for validation of PCR assays for the detection of the *tet*A and *sul*1 gene.
