# Supplementary material for: High dietary zinc supplementation increases the occurrence of tetracycline and sulfonamide resistance genes in the intestine of weaned pigs
Source: Gut Pathog. 2015 Aug 26;7:23. doi: 10.1186/s13099-015-0071-3 (PMC4551370; doi:10.1186/s13099-015-0071-3)
Supplement: Supplementary file 1 — Additional file 1. Strains used for validation of PCR assays for the detection of the tetA and sul1 gene. [file 13099_2015_71_MOESM1_ESM.docx]

Supplemental Table 1: Strains used for validation of PCR assays for the detection of the *tet*A and *sul*1 gene

| Species | Strain origin^1^ | *tet*A^*^ | *sul*1 |
| --- | --- | --- | --- |
| *Bacillus cereus* | DSM 31 | - | - |
| *Citrobacter freundii* | DSM 30039 | + | - |
| *Corynebacterium glutamicum* | DSM 20300 | - | - |
| *Enterobacter cloacae* | DSM 46348 | - | + |
| *Enterococcus faecium* | DSM 2918 | - | - |
| *Erwinia billingiae* | DSM 17872 | - | - |
| *Escherichia coli* | IMT 18570 | + | - |
| *Escherichia coli* | IMT 13211 | + | + |
| *Escherichia coli* | DSM 2840 | - | - |
| *Escherichia coli* | DSM 10233 | - | + |
| *Klebsiella pneumoniae* | DSM 16609 | + | + |
| *Klebsiella* sp. | IMT17506 | - | + |
| *Klebsiella* sp. | IMT17441 | - | + |
| *Lactobacillus acidophilus* | DSM 20079 | - | - |
| *Lactobacillus amylovorus* | DSM 20531 | - | - |
| *Salmonella enterica* subsp. *Enterica* | DSM 9898 | + | + |
| *Streptococcus suis* | DSM 9682 | - | - |

^*^ = - = negative; + positive

^1^ = DSM = Deutsche Sammlung von Mikroorganismen und Zellkulturen, Braunschweig, Germany; IMT = Institute of Microbiology and Epizootics, Freie Universität Berlin, Berlin, Germany

Supplemental Table 2a: Time dependent effect of *tet*A copy number development in weaned pigs fed different concentrations of dietary ZnO [log copy number *tet*A/ gram wet weight]

| **Sampling day^*^** | **Low ZnO** | **Intermediate ZnO** | **High ZnO** |
| --- | --- | --- | --- |
| 6 | 4.92(x0.81) | 4.95(x0.66) | 5.07(x0.32)^a^ |
| 13 | 4.74(x0.58) | 4.85(x0.71) | 5.23(x0.60)^ab^ |
| 20 | 4.68(x0.66) | 5.01(x0.48) | 5.48(x0.66)^b^ |
| 27 | 4.80(x0.51) | 4.94(x0.50) | 5.38(x0.57)^b^ |

^*^ = Combined data from all intestinal sites

^a,b^ = significantly different within a column (Mann-Whitney-U test, p<0.05)

Supplemental Table 2b: Time dependent effect of *sul*1 copy number development in weaned pigs fed different concentrations of dietary ZnO [log copy number *sul*1/ gram wet weight]

| **Sampling day^*^** | **Low ZnO** | **Intermediate ZnO** | **High ZnO** |
| --- | --- | --- | --- |
| 6 | 5.52(x0.59) | 5.58(x0.67) | 5.67(x0.21)^a^ |
| 13 | 5.48(x0.67) | 5.40(x0.73) | 5.99(x0.55)^b^ |
| 20 | 5.31(x0.65) | 5.49(x0.63) | 5.86(x0.45)^b^ |
| 27 | 5.41(x0.63) | 5.53(x0.73) | 5.78(x0.59)^ab^ |

^*^ = Combined data from all intestinal sites

^a,b^ = significantly different within a column (Mann-Whitney-U test, p<0.05)
